# Lost in Translation: Investigating Barriers to Older Male Participation in Intergenerational Research

**DOI:** 10.1002/alz70858_105346

**Published:** 2025-12-25

**Authors:** Katya T. Numbers, Nicole Ee, Maoling Lim, Suraj Samtani, Ruth Peters, Amy Sparks, Kaarin J. Anstey

**Affiliations:** ^1^ Centre for Healthy Brain Ageing (CHeBA), University of New South Wales, UNSW Sydney, NSW, Australia; ^2^ The George Institute for Global Health, Sydney, NSW, Australia; ^3^ University of New South Wales, Sydney, NSW, Australia; ^4^ Neuroscience Research Australia, Sydney, NSW, Australia; ^5^ University of New South Wales, Kensington, NSW, Australia; ^6^ Centre for Healthy Brain Ageing (CHeBA), UNSW Sydney, Sydney, NSW, Australia; ^7^ The George Institute for Global Health, University of New South Wales, Imperial College London, Sydney, NSW, Australia; ^8^ UNSW Ageing Futures Institute, Syndey, NSW, Australia; ^9^ Neuroscience Research Australia (NeuRA), Sydney, NSW, Australia

## Abstract

**Background:**

Intergenerational programs reduce dementia risk and promote healthy ageing through social engagement, reduced loneliness, and cognitive stimulation. However, older men remain underrepresented, limiting the generalisability of findings and development of tailored interventions. This study explored barriers preventing male participation and strategies to improve engagement.

**Method:**

Three focus groups with eight Australian men aged 60 and older explored participation barriers, recruitment reactions, and program preferences. Sessions were recorded, transcribed, and analysed using Speak Ai. A complementary survey with 220 participants (mean age = 72.89, SD = 5.26 years) evaluated mock recruitment materials with varying age and gender tones. Participants rated their willingness to participate and provided preferences for study formats, group sizes, and age ranges.

**Result:**

Focus groups revealed that traditional masculinity norms were a key barrier. Participants viewed intergenerational programs as caregiving roles conflicting with their identity. Abstract terms like ‘relationships’ were unrelatable, while action‐oriented phrases such as ‘mentoring’ and ‘skill‐sharing’ resonated. Concerns included child safety, societal stigma, and police check requirements. Structured, skill‐based activities like coaching and physical tasks were preferred.

Survey findings supported these themes. Gender‐neutral (18.6%) and feminine (18.2%) titles were more appealing than masculine titles (14.5%). Age‐positive titles (18.6%) were favoured over age‐neutral (13.6%) and age‐negative (16.4%) messaging. Research benefits (45%) were more persuasive than societal (30.5%) or personal benefits (5%). Neutral gendered blurbs (48.2%) and age‐positive blurbs (35.5%) attracted the most interest. Participants overwhelmingly preferred small groups of 2–10 people (71.4%) and mixed‐gender settings (89.1%). In‐person formats (57.7%) were favoured over online‐only (26.4%). Interaction with primary school‐aged children (50%) was preferred to younger or older age groups.

**Conclusion:**

These findings highlight the need for recruitment strategies that resonate with older men's values. Relatable, action‐focused language, structured activities, and transparent safety protocols can enhance engagement, improving inclusivity in intergenerational research and supporting efforts to reduce dementia risk and promote healthy ageing.

